# Improving the cost-effectiveness of IRS with climate informed health surveillance systems

**DOI:** 10.1186/1475-2875-7-263

**Published:** 2008-12-24

**Authors:** Eve Worrall, Stephen J Connor, Madeleine C Thomson

**Affiliations:** 1 Liverpool Associates in Tropical Health, 15-17 Seymour Street, Seymour Terrace, Liverpool, UK; 2 International Research Institute for Climate & Society, The Earth Institute at Columbia University, New York, NY, USA

## Abstract

**Background:**

This paper examines how the cost-effectiveness of IRS varies depending on the severity of transmission and level of programme coverage and how efficiency could be improved by incorporating climate information into decision making for malaria control programmes as part of an integrated Malaria Early Warning and Response System (MEWS).

**Methods:**

A climate driven model of malaria transmission was used to simulate cost-effectiveness of alternative IRS coverage levels over six epidemic and non-epidemic years. Decision rules for a potential MEWS system that triggers different IRS coverage are described. The average and marginal cost per case averted with baseline IRS coverage (24%) and under varying IRS coverage levels (50%, 75% and 100%) were calculated.

**Results:**

Average cost-effectiveness of 24% coverage varies dramatically between years, from US$108 per case prevented in low transmission to US$0.42 in epidemic years. Similarly for higher coverage (24–100%) cost per case prevented is far higher in low than high transmission years ($108–$267 to $0.88–$2.26).

**Discussion:**

Efficiency and health benefit gains could be achieved by implementing MEWS that provides timely, accurate information. Evidence from southern Africa, (especially Botswana) supports this.

**Conclusion:**

Advance knowledge of transmission severity can help managers make coverage decisions which optimise resource use and exploit efficiency gains if a fully integrated MEWS is in place alongside a health system with sufficient flexibility to modify control plans in response to information. More countries and programmes should be supported to use the best available evidence and science to integrate climate informed MEWS into decision making within malaria control programmes.

## Background

Quantifying the burden of malaria remains a major challenge as many infections may be asymptomatic, inadequate diagnosis and reporting and the fact that a majority of febrile cases do not reach the formal health system make estimation imprecise. Recent estimates espouse the figures of 300–600 million infections and 1–3 million deaths per year [1]. Less contentious is the fact that Africa bears the brunt of the malaria burden, with estimates suggesting greater than 60% of the worlds clinical cases and more than 90% of the worlds malaria deaths [2].

Africa's population is estimated to pass 1 billion people by 2010 [3]. The current goal of the Global Malaria Programme and the Roll Back Malaria Partnership is to "halve the malaria burden by 2010" by focussing on treatment, prevention and epidemic response[4]. Africa is not yet on track to achieve this goal [5].

It is estimated that 615 million Africans live in endemic regions, where those most at risk are children under 5 and pregnant women, non-pregnant women and adult males are protected, to varying degrees, by acquired immunity. There are a further estimated 125 million people at risk from epidemic malaria in sub Saharan Africa [6]. By definition in epidemic prone areas, transmission intensity (usually indicated by the entomological inoculation rate, or EIR) is insufficient for the population to develop acquired immunity; therefore this entire population of 125 million people is at risk of severe morbidity and mortality from malaria [7]. People living in malaria endemic regions acquire immunity to malaria through natural exposure to malaria parasites. This naturally acquired immunity is protective against parasites and clinical disease but it only results after continued exposure from multiple infections over time. The transmission intensity influences the course of development of clinical and parasitic immunity. In areas of intense transmission, young children bear the burden of malaria, but as they grow older they build up acquired immunity. In areas of low endemicity both children and adults suffer disease and high parasitaemia because exposure is less [8].

Pregnant women are more susceptible than non-pregnant women to malaria because of a combination of immunological and hormonal changes associated with pregnancy, and with the unique ability of certain variants of infected erythrocyts to sequester in the placenta [9]. Proportionally more pregnant women are at risk in epidemic settings because in high transmission areas, multi-gravidae are partially protected from adverse effects and only primigravidae are indisputably at greater risk of infection than non-pregnant women [10]. The clinical features of malaria infection during pregnancy also vary by epidemiologic setting. Severe disease (such as cerebral malaria and respiratory distress syndrome) is a more common characteristic in low and unstable malaria transmission areas, whereas pregnant women in high transmission areas rarely experience cerebral malaria but instead have more frequent exposure to malaria leading to severe anaemia [11]. In all areas, malaria in pregnancy is responsible for low birth weight (LBW) deliveries [12].

In epidemic prone areas malaria control interventions aim to protect the entire population for mortality, whereas in endemic areas, interventions only afford protection to vulnerable sub-population groups such as young children and pregnant women from mortality and the general population from morbidity.

Approximately 20% of sub-Saharan Africa's population, under five's and pregnant women, living in endemic malaria areas are subject to malaria as a major life threatening disease (Africa Malaria Report: WHO-AFRO 2006). This is roughly equivalent in numbers to the estimated 125 million people at risk of severe disease and death from malaria epidemics [7]. However, although the population at risk is roughly equivalent in endemic and non endemic areas, in the former the risk is constant from one year to the next whereas in epidemic prone areas the risk may only be high in certain years. Hence the temporal element of risk needs to be considered. Thus endemic and epidemic malaria require different approaches to control and prevention. Endemic malaria requires ongoing measures whereas successful control of epidemic malaria relies on measures being applied in the right place at the right time, this is even more important when resources are scarce [13].

The global malaria strategy calls for sustained commitment to address malaria epidemics [14], but there has been less focus on epidemics relative to other strategic areas of intervention such as scaling up of ITN use.

Recently there has been a renewed emphasis on malaria vector control with indoor residual spraying (IRS), especially under the US Government's Presidential Malaria Initiative (PMI). PMI is supporting enhanced malaria control in fifteen African countries, many of which are or will be using IRS as part of a package of malaria control. A growing body of evidence shows that IRS and ITNs (and more recently LLINs) are highly cost-effective malaria control interventions [15,16]. More recently focus has shifted towards examining the relative cost-effectiveness of these two interventions in comparison to each other [17]. However, there is still a dearth of evidence on the cost-effectiveness of malaria control interventions in epidemic prone areas [6] and economic evaluations of interventions in epidemic or seasonal transmission areas commonly fail to account for key differences in assessing malaria control interventions in epidemic as opposed to endemic settings. A previous paper has shown how the effectiveness of IRS (in terms of cases prevented) varies according to the magnitude of transmission each year, as well as the timing of IRS in relation to the transmission season [18].

This paper examines how the cost-effectiveness of IRS varies depending on the severity of transmission and level of coverage. It also investigates how efficiency could be improved by incorporating climate information into the decision making process of malaria control programmes as part of an integrated Malaria Early Warning and Response System (MEWS) [19]. Put another way this study aims to show how more health impact can be obtained through use of the same or similar resources if climate information was used to inform programme decisions.

Firstly, available evidence on the cost and cost-effectiveness of IRS in sub-Saharan Africa is reviewed to establish what is already known about the key drivers of cost and cost-effectiveness of IRS and highlight limitations of existing studies. Secondly, novel cost data on an operational IRS programme from Zimbabwe is presented. Thirdly, investigation is made into how cost-effectiveness and marginal cost-effectiveness of IRS varies depending on severity of transmission and simulated level of IRS programme coverage. Finally, a discussion focuses on how the cost-effectiveness of IRS and other malaria control interventions can be improved through the incorporation of climate information into malaria control programming decisions.

### Cost of indoor residual spraying

Table 1 summarizes the results of studies on the cost (and cost-effectiveness) of IRS in sub-Saharan Africa. Only four published studies were located on the cost-effectiveness of actual IRS programmes in sub-Saharan Africa:

**Table 1:** Economic cost (in 2000US$*) and CE of IRS in sub-Saharan Africa

**Study (number) and Location** [costs reported in original study]	**Intervention description and scale**	**Unit Cost per year^†^** (rounded to nearest cent)	Cost-effectiveness (rounded to nearest $)
Study (1) Tanzania [20]	• IRS one round per year • Lambda chyhalothrin (Icon) • 1000 people covered	$2.45 per person protected	No Data

Study (2) Highland Kenya [2000 $]	• IRS one round per year • Lambda cyhalothrin (Icon) • 1752 people covered	$0.88 per person protected [22]	$9 per infection case prevented [21]

Study (3) KwaZulu Natal, S Africa [23] [1999 Rand and $]	• IRS one round per year • Deltamethrin • 26,703 people covered	$4.93 per person protected	No Data

Study (4) LSDI, Mozambique [2000 $]	• IRS two rounds per year • FICAM and Propoxur • 251,918 people covered Zone 1: 1 round FICAM, 1 round Propoxur (71047 people) Zone 1A: 2 rounds FICAM (180871 people)	$4.82 per person protected Zone 1: $3.48 per person protected Zone 1A: $2.16 per person protected	$28 per clinical case averted $29 per infection case prevented

Study (5) Reanalysis of Kwa-Zulu Natal, South Africa [17] [$2005]	• IRS one round per year • Deltamethrin • 26,703 people covered	$4.27 per person protected	$596 per death averted $23 per DALY averted‡
		
		$30.35 cost per under five protected	$5518 per under five death averted $167 per under five DALY averted

Study (6) Reanalysis of LSDI, Mozambique** [17] [$2005]	• IRS two rounds per year • FICAM and Propoxur • 251,918 people covered	$4.94 per person protected	$897 per death averted $27 per DALY averted
		
		$27.40 per under five protected	$4981 per death averted $151 per DALY averted
	
	• IRS one round per year • Lambda cyhalothrin (Icon)	$4.34–7.67 per under five protected [26]	$12–22 per under five DALY averted [25]
	
	• IRS two rounds per year • Lambda cyhalothrin (Icon)	$8.69–15.34 per under five protected [26]	$24–44 per under five DALY averted [25]

*Converted from original source by author where necessary using US government GDP price deflator: U.S. Bureau of Economic Analysis (BEA) (2008). U.S. Implicit Price Deflators for Gross Domestic Product [On-line]. Available:

^†^Unit cost per year = Total annual costs divided by population protected by intervention

^‡^Examines only child deaths but allocates costs to child DALYs averted based on proportion of children in population

1. A comparison of IRS and ITNs in Tanzania [20]

2. A comparison of IRS and ITNs in highland Kenya [21,22]f

3. A comparison of IRS and ITNs in KwaZulu-Natal, South Africa [23]

4. An evaluation of IRS in Mozambique [24]

Further studies of relevance are:

5. A reanalysis of the South African KwaZulu-Natal study [17]

6. A reanalysis of the Mozambique study [17]

7. A comprehensive modelling study comparing the cost-effectiveness of a variety of malaria control interventions [25,26].

The studies show that the unit cost per year of IRS is between $0.88 (study 2 highland Kenya) and $30.35 (study 5 reanalysis of Kwazulu-Natal). However, there are some methodological and real differences which explain these differentials in costs. For example some studies only valued the protection afforded to children whereas others included the whole population. Costing methodology may also have differed (for example what is included or excluded). The type and price of insecticide used will also affect cost (and effectiveness) of programmes. Other factors which may cause variation in programme cost are the programme structure, population density, geographical area and topography of where the spray programme took place, as well as the scale and efficiency of the programme. See Additional file 1 for a more detailed discussion of these comparability issues.

### Cost-effectiveness

Table 1 shows the cost-effectiveness of IRS programmes for the 7 studies (where available). The studies are not consistent in the effectiveness indicator used. Studies 2 and 4 provide a cost per infection case prevented ($9 and $29 respectively). Study 3 is a marginal cost analysis comparing ITNs to IRS and as such does not give the cost-effectiveness of IRS alone. Study 5 and 6 carried out additional analysis on study 3 and 4 to come up with a cost per death and Disability Adjusted Life Year (DALY, combines the morbidity and mortality indicators into a single unit) averted. In the Kwazulu-Natal reanalysis (study 5) the cost per death averted was $596 compared to $897 in Mozambique (study 6). The cost per DALY averted in these two studies was similar at $23 and $27 respectively, a similar range to the modelling study (7) which estimated a cost per DALY averted of between $12–22 for a single spray round and between $24–44 for two spray rounds.

That these results are fairly consistent (where comparable) in terms of cost per infection case, death and DALY averted, is encouraging. It is also important to point out that the cost-effectiveness of IRS is attractive based on these results [27].

A critical factor overlooked by all the existing evaluations of IRS reviewed here is the climate related inter-annual or spatial variation in malaria transmission. Epidemics are often the health manifestation of weather or climate anomalies (which have resulted in extensive flooding or an extended transmission season) which directly increase the hazard to the population (in this case transmission intensity)

The level of risk of any population may be understood as a function of the hazard and the population's vulnerability to that hazard (in this case population immunity and socio-economic factors).

In epidemic areas IRS protects the population against a hazard which may vary from year to year according to the climate. Assessments of the impact of IRS and other anti-malaria interventions which do not take into account the climate risk may introduce serious bias into the results [28]

Studies on the impact of IRS which do not control for climate variation cannot be sure if it is the IRS, or for example drought, which is responsible for reductions in malaria incidence; in many parts of the world droughts are a good predictor of control 'success'!

Of the empirical studies considered here, 2, 3 and 4 were carried out in areas of seasonal and epidemic prone malaria transmission, yet none of them consider the impact of climate variability and therefore potentially overestimate or underestimate the benefits of IRS because of spatial or temporal variations between the intervention site and/or year and the control.

## Methods

The economic costs of the IRS programme in Hwange District, of Zimbabwe were captured for a twelve month period aligned with the 1998/99 malaria transmission season using standard economic costing methodology [29]. Full details of the study area, IRS programme, costing methods and results are given in Additional file 2.

Using the same model presented in a previous paper [30], the unit cost data was combined with estimates of the effectiveness of IRS over a six year period to examine how the average and marginal cost-effectiveness of a programme varies depending on (i) the level of spray coverage achieved and (ii) the severity of transmission. The model was then used to simulate the number of cases that would occur given five alternative levels of spray coverage, effective from January each year. The coverage levels examined were 0%, 24%, 50%, 75% and 100%.

24% coverage is the actual level of coverage achieved overall in Hwange, District this is because the IRS programme targeted administrative units smaller than districts, achieving high levels of coverage (80–95%) in these sub-units but an average coverage level of 24% throughout the District. Hence what our coverage levels refer to is the percentage of the district targeted for IRS with the implicit assumption that the level of coverage achieved is sufficiently high (> 85%) for the IRS to achieve impact.

For each year and level of spray coverage the total cost of the spray programme was calculated, the number of cases prevented and the average cost per case prevented compared to the no coverage (0%) scenario. Next the marginal cost per case prevented of additional coverage levels compared to the baseline (24%) scenario was calculated. Full details and results of simulations on cases and cases prevented can be found in [30].

In order to examine the impact of severity of transmission, each year was categorized in our dataset as low, medium, high or epidemic depending on the number of simulated cases with no intervention) (0–50,000; 50,001–100,000; 100,001–150,000 and 150,001–200,000 respectively) as shown in columns 1 – 3 of Table 2.

**Table 2:** Transmission season categories and implied IRS coverage levels

1	2	3	4
Cases occurring with no intervention	Severity of transmission (actual or predicted)	Years in this category	Implied IRS Coverage Level

0–50,00	Low transmission	1993, 1994	24%

50,001–100,000	Medium transmission	1995	50%

100,001–150,000	High transmission	1997 1998	75%

150,001–200,000	Epidemic	1996	100%

Next the potential efficiency implications of a MEWS which would allow programme managers to choose the level of IRS programme coverage in advance based on information about the likely severity of the forthcoming malaria transmission season was examined. It was assumed that the forecast would influence decision making regarding IRS programme coverage, as shown in columns 2 and 4 of Table 2, as part of a MEWS. This was labelled the "implied level of coverage", i.e. the level of coverage that the MEWS indicated would be appropriate given predicted transmission potential each year. Note that there are no years where 0% coverage would be recommended and alternatives are being compared to the baseline level of coverage (24%) hence the key variable of interest here is marginal (as opposed to average) cost-effectiveness.

## Results

### IRS programme costs

The costs of the Hwange District IRS programme are summarized in Table 3. The total cost was US$47,000 with chemical costs forming the vast majority (81%) of programme costs followed by equipment costs (11%) and labour costs (4%).

**Table 3:** Cost of Hwange District IRS programme (US$)

Cost Item	US$	% of Total Cost
Chemicals	38023	81

Labour (excluding Community Education)	1947	4

Transport	1459	3

Equipment	5118	11

Community Education	518	1

TOTAL	47065	100

The number of structures sprayed in Hwange district was 24,236 and the estimated population covered was 39,416 (Source: Hwange District Environmental Health Office and Matabeleland North Provincial Environmental Health Office). The cost per person protected by the spray programme was therefore $1.01 in 2000 US$.

This result is comparable with those in Table 1. although at the low end. One reason for this could be the lack of inclusion of all central level costs however it is also likely to be due to the fact that this was an operational programme being carried out by the Government of Zimbabwe with no external international assistance which can increase programme costs. An operational programme is also far from the "optimal" programme which would be costed under a comprehensive modelling approach (e.g. study 7) which seeks to include all required inputs. For example, in the Hwange programme, very little resources were devoted to supervision and community sensitisation, and none to monitoring and evaluation.

### Average cost-effectiveness

The average cost-effectiveness (Table 4) of the baseline level of coverage (compared to 0% coverage) varies dramatically between years, from US$108 per case prevented in 1993 (a low transmission year where IRS can potentially prevent relatively fewer cases) to US$0.42 in 1996, the epidemic year where the potential gains from effective high coverage IRS are far higher. The results also show the variation in range of average cost-effectiveness of different coverage levels depending on severity of transmission. In the lowest transmission year (1993) the range for 24%–100% coverage is between $108–$267 per case prevented, whereas in the epidemic year (1996) the range is lower and narrower at between $0.88–$2.26 per case prevented. Overall the results show that it is more cost-effective to intervene in high transmission years and that even at 100% coverage the cost per case prevented is still below $10 in all but the low transmission years (1993 and 1994).

**Table 4:** Average cost per case prevented (compared to 0%) of alternative coverage levels (US$)

IRS coverage level				
Year	24% (Baseline)	50%	75%	100%
1993	108.12	153.99	206.84	267.15

1994	4.23	6.01	8.07	10.41

1995	0.87	1.33	1.83	2.37

1996	0.42	0.57	0.72	0.91

1997	0.63	0.97	1.31	1.69

1998	0.88	1.36	1.78	2.26

### Marginal cost-effectiveness

The marginal cost-effectiveness results show how much it would cost to prevent additional cases by increasing the IRS coverage level from the baseline (24%) to 50%, 75% or 100% (Table 5). In medium, high or epidemic transmission years the marginal cost per case prevented is never more than $5.19 even up to coverage levels of 100%. However, in 1993 (low transmission) the marginal cost-effectiveness of 100% coverage was approximately $500 per case prevented. This illustrates that relative to other years, increased levels of spray coverage in low transmission years are not an efficient use of resources.

**Table 5:** Marginal Cost per Case prevented (compared to baseline, 24%) of alternative coverage levels

IRS coverage level			
Year	50%	75%	100%
1993	253.13	362.66	498.86

1994	9.84	14.05	19.29

1995	2.60	3.77	5.19

1996	0.85	1.08	1.42

1997	1.94	2.67	3.60

1998	2.73	3.42	4.49

### Potential efficiency gains

Next the efficiency implications of an IRS programme where the implied level of coverage as implied by the MEWS forecast was sprayed each year were examined. In 1993 and 1994 (low transmission years) the system would dictate 24% (baseline) coverage. At these coverage levels the average and marginal cost per case prevented would be $108.12 and $4.23 respectively (Table 6). This system would ensure that as discussed above, relatively inefficient high levels of coverage would be avoided in low transmission years. In the epidemic transmission year (1996) 100% spray coverage would still only cost an average and marginal cost of $0.91 and $1.42 respectively, clearly if the programme is affordable these ratios are highly attractive.

**Table 6:** Average and Marginal Cost-effectiveness of MEWS informed decisions

Year	MEWS prediction	Implied IRS Coverage Level	Average cost-effectiveness at implied coverage level ($)	Marginal cost-effectiveness at implied coverage level ($)
1993	Low	24%	108.12	-

1994	Low	24%	4.23	-

1995	Medium	50%	1.33	3.77

1996	Epidemic	100%	0.91	1.42

1997	High	75%	1.31	2.67

1998	High	75%	1.36	2.73

### Study limitations

The unit cost estimate of $1.01 per person protected with IRS was used for all years to avoid introducing additional variation into the analysis that would stop us from observing the impact of changes in the two variables of interest (severity and coverage). A key limitation of using a fixed unit cost per person protected is that it assumes constant returns to scale with spray programme coverage. This is unlikely to reflect the reality where there are likely to be economies of scale up to point where diseconomies set in and the costs of reaching the last few individuals increase. Neither these data nor the literature gives any clear indication regarding the impact of scale on unit cost of spray programmes. The impact of this on the results in this study is likely to be constrained because in all IRS programmes the cost of the insecticide is a large proportion of total costs and hence the ability to achieve significant economies of scale is limited by the relatively fixed unit cost of insecticides. In addition, the coverage level of 100% used in this analysis is used for convenience only, in reality programme coverage of 100% is not operationally achievable and so resources would not be used to reach the last, most expensive person/households.

Using a system where increased levels of coverage are triggered by a forecast of epidemic risk is likely to cost more, especially if the cost of increased coverage is not fully offset by reduced coverage in low transmission years. However, this analysis has shown that the value of that protection in terms of health benefit (cases prevented) is greater in high transmission years. This additional expense must therefore be offset against the additional health benefits and financial savings made through greater efficiency of interventions in high transmission and epidemic years. This analysis has examined the cost from the provider perspective but has not included any potential cost savings to the health system of preventing malaria cases. Preventing cases occurring will save on drug consumption and possibly staff time within the health system, subject to accurate malaria diagnosis and flexibility in the system. There may also be savings in out of pocket expenditure and reduced opportunity cost from households associated with preventing cases.

In the current analysis, there are four possible levels of transmission (low, medium, high and epidemic) detected by the MEWS, that trigger a decision to deliver four alternative coverage levels. In reality systems may not be this sophisticated, but this has just been for the purposes of the analysis to obtain more data points. In reality a programme may choose only to have three or even two different coverage level triggers and decisions to simplify the system, but there are still potential efficiency gains to be made. For example in Botswana, where case surveillance is used as part of a MEWS there are three case thresholds which have distinct predetermined actions; deploying additional medical staff to the area, deploying mobile treatment teams and declaring a disaster to trigger disaster response plan [13]. Decisions also need a geographical element and the results of this study would equally apply to this element. For example, a decision to target a relatively high transmission area would result in a more efficient use of resources than spraying all districts with the same level of coverage regardless of transmission intensity.

The exact nature of the decision matrix (as illustrated in Table 2) would have to be developed by individual programmes depending on a number of factors such as available resources, country priorities, epidemiology and coverage of health services. In the example presented above it might be appropriate to have not sprayed at all in 1993 since the number of cases occurring with no intervention and therefore the potential health benefit of any level of IRS coverage would be very low, this is reflected in the high average cost per case prevented at 24% coverage of $108.12. A decision not to spray at all however, would have to be balanced by a high degree of certainty that the forecast was correct.

## Discussion

For this type of system to be of practical use (and reap the hypothetical efficiency and health benefit gains presented in this paper) there are two interrelated key factors which need to be considered. The first is the accuracy (ability to predict malaria season severity) of the information and the second is the timeliness of the information, especially in relation to health services capacity to respond. The present analysis assumes that the MEWS is accurate (does not predict false epidemics or miss real epidemics) and that the information is available sufficiently early for programme managers to use it as a basis to make IRS programme coverage decisions. WHO has, since 2002, promoted a framework for malaria early warning in which four components are operating [31]. The components are: 1) vulnerability monitoring; 2) seasonal climate forecasting; 3) environmental monitoring and 4) sentinel case surveillance; each informing elements of control planning, preparedness and response.

Vulnerability monitoring of factors such as HIV AIDS incidence, drug resistance and food insecurity helps keep track of factors which increase the severity of disease outcome should an epidemic occur. Seasonal climate forecasts provide advance warning of the potential for malaria epidemics to occur. Environmental monitoring, e.g. of rainfall can be used to monitor the accuracy seasonal climate forecasts, yet it still offers some lead time to inform programme decisions. Sentinel case surveillance data is of paramount importance in early detection of epidemics but may come too late to trigger a full preventive response. The WHO framework to malaria early warning has been adopted in a number of epidemic prone countries in Southern Africa [32]. In Botswana an analysis of national confirmed malaria incidence over 20 years revealed that year to year variation in rainfall could account for the year to year variations in incidence once long term vulnerability changes had been taken into account [33].

Furthermore seasonal climate forecasts issued in November, four to five months prior to the peak malaria season, were found to accurately predict five low malaria transmission years and four out of five high transmission years in Botswana [34].

Of the southern African countries (Botswana, Madagascar, Mozambique, Namibia, South Africa, Swaziland and Zimbabwe) involved in these efforts, Botswana has perhaps the most advanced and well resourced system. There is evidence that in a recent wet year following several dry years (2005–06 malaria season), cases were ten times lower in Botswana than in a previous year with similar conditions (1996–97). In Zimbabwe, where political and economic constraints have hampered efforts to implement MEWS and malaria control more generally, cases were down by half. MEWS may have contributed to these successes but changes in other factors (e.g. availability of drugs and increased ITN use) will also have had an effect [13].

## Conclusion

For practical reasons, programme managers are not likely to be interested in such detailed analysis of average and marginal cost per case prevented. However, on a broad operational level the results clearly show that compared to spraying the same level of coverage each year, programme managers could ensure a more effective and efficient use of resources by using information on likely transmission severity to inform IRS programme coverage. Accurate advance knowledge of the severity of the transmission season could help managers make coverage decisions which optimize resource use and exploit efficiency gains afforded by the greater effectiveness of IRS in high transmission seasons. They can also avoid using resources unnecessarily in a low transmission year.

The efficiency gains examined in this paper could only be realized if a fully integrated MEWS was in place within a health system with sufficient flexibility to modify control plans in response to forecasts. This is the case in Botswana where it is likely that some efficiency gains and greater health benefits are being realized, if not actually quantified. Increasing the use of MEWS and flexible response systems further, especially in relation to IRS programme planning and decision making, has the potential to ensure malaria control programme decisions are more efficient and critically have greater health impact.

Increased resources are available for both malaria control and IRS in particular. However, resources are still scarce in relation to the burden of malaria. There is thus a need to ensure resources are used as efficiently as possible to generate maximum health benefit. We have the scientific understanding to predict malaria transmission using climate and other indicators [34]. There is also increasing evidence and examples of the use of climate based early warning systems being used as a decision making tool in malaria control programmes [13,32]. More countries and programmes should be supported to use the best available evidence and scientific know how to integrate climate informed MEWS into decision making within malaria control programmes. The current focus on climate change adaptation and improved management of climate sensitive risk in national development sectors offers opportunities for Ministries of Health to request greater engagement with their national counterparts in climate and weather services. There are currently only a few countries pioneering this approach, however national and international development donors are seeking to invest strongly in improving climate risk management in coming years [13,35]

## Competing interests

The authors declare that they have no competing interests.

## Authors' contributions

MT, SC and EW contributed to the conception and design of the study. EW carried out acquisition of data and MC, SC and EW carried out analysis and interpretation of data. EW drafted the manuscript and SC and MC have revised it critically for important intellectual content. All authors have given final approval of the version to be published.

## Supplementary Material

Additional File 1 **IRS cost effectiveness comparability issues**. Discussion addressing challenges of comparing IRS cost and cost effectiveness data from different settings and methods. Click here for file

Additional File 2 **Cost of Hwange District, Zimbabwe IRS programme**. Detailed results of Hwange district IRS programme costing analysis including unit cost data. Click here for file
