# Supplementary material for: Improving the cost-effectiveness of IRS with climate informed health surveillance systems
Source: Malar J. 2008 Dec 24;7:263. doi: 10.1186/1475-2875-7-263 (PMC2639594; doi:10.1186/1475-2875-7-263)
Supplement: Additional File 1 — IRS cost effectiveness comparability issues. Discussion addressing challenges of comparing IRS cost and cost effectiveness data from different settings and methods. [file 1475-2875-7-263-S1.docx]

# Additional File 1: IRS cost effectiveness comparability issues

### Population protection

Studies 1-4 present costs per person protected per year, and study 7 presents cost per under five child protected per year calculated by adjusting total costs by the proportion of children in the population (hence this is comparable to cost per person protected in 1-4). Studies 5 and 6 report both cost per person protected ($4.27 and $4.94 for Kwazulu-Natal and Mozambique respectively) and per under-five child protected ($30.35 and $27.40 respectively) illustrating how altering the unit of cost can dramatically affect the unit costs (if costs are not adjusted to account for proportion of children in the population as in 7). The decision whether to divide total costs by the under five or total population should depend on the purpose of the study but more importantly on the epidemiology of malaria in the area being studied. In epidemic prone areas all age groups are at risk of malaria morbidity and mortality, and so it is important to capture the benefits accruing from IRS to all age groups. Given that highland Kenya, Kwazulu-Natal and Mozambique are areas which exhibit highly seasonal transmission with epidemic potential, it is more appropriate to use the cost per person protected since adults as well as children will have partial immunity and thus benefit from protection from malaria afforded by IRS. Already this shows how the potential cost effectiveness of IRS is greater in epidemic prone areas where a larger proportion of the population benefit from protection afforded.

### Choice and price of insecticide

Another factor which could be assumed to have an impact on costs of IRS is the choice of insecticide used. In most IRS programmes the cost of insecticide will be a major contributor to the cost of the programme and this means that cost of insecticide will have a reasonably large impact on cost per person protected (though other programme design factors obviously influence this). Insecticides have different residual effects (useful life), target dosage (amount of active ingredient required to be delivered to house wall) and prices. Therefore, comparison of the costs of alternative insecticides is not straightforward. Table 7 shows a variety of pricing information for DDT and each of the different insecticides used in studies 1-7 and supplemented by data from another study [1].

### Other factors influencing cost

The number of spray rounds per year is also likely to influence cost per person protected. The number of rounds required will depend on the length of the transmission season and the residual life of the insecticide. Studies 1-3 and 5 (Tanzania, Kenya, Kwazulu-Natal and reanalysis of Kwazulu-Natal), have one round per year, but study 4 and 5 (Mozambique and reanalysis) have two rounds per year. However, the cost per person protected in Kwazulu-Natal and Mozambique are similar. This could be due to the relative proportion of fixed to variable costs. A programme with high fixed costs (e.g. salaries and capital costs) will incur high costs regardless of the number of spray rounds carried out. Whereas a programme with relatively low fixed costs will see marked increase in costs as additional spray rounds are carried out. The modelling study (vi) examines the cost of one and two rounds per year and finds that two rounds are twice as costly as a single round. However, since this result is not borne out by the empirical studies it could be a weakness of the modelling approach.

A number of other factors not considered in detail here could also explain the variation in cost per person protected between these studies including the costing methodology (for example what is included excluded), the programme structure, population density, geographical area and topography of where the spray programme took place, as well as the scale and efficiency of the programme.

# Table 7 Cost comparison of alternative insecticides for IRS

| **A** | **B** | **C** | **D** | **E** | **F** | **G** | **H** |
| --- | --- | --- | --- | --- | --- | --- | --- |
| Study (number) using this insecticide | Insecticide Type | Insecticide Name and Brand Name | Cost per KG from South African suppliers year 2000 (US$2000) [2] | Cost per KG active ingredient, various sources, in US$1995 [3] | Average price quoted from various sources in 1998-9 in US$1999 [4] | | Authors calculation |
|  |  |  |  |  | Cost per KG/Litre | Cost per house per six months | Ratio of price relative to ICON |
| None | Organo-chlorine | DDT | 5.3 | 4.67-6.04 | 5.63 | 3 | 0.67 |
| Tanzania (1)  Kenya (2)  Modelling (7) | Pyrethroid | Lambda cyhalothrin (ICON) | 66 | 700 | 75.2 | 4.51 | 1.00 |
| Mozambique (4)  Mozambique reanalysis (6) | Carbamate | Bendiocarb (FICAM) | 72.5 | Not given | 80 | 8 | 1.77 |
| Mozambique (4)  Mozambique reanalysis (6) | Carbamate | Propoxur (Baygon) | 25 | Not given | 9.3 | 56 | 12.42 |
| Kwazulu Natal (3)  Kwazulu Natal reanalysis (5) | Pyrethroid | Deltamethrin (K-Othrine) | Not given | 684.4-895.2 | 20 | 8 | 1.77 |

1. Walker K: **Cost-comparison of DDT and alternative insecticides for malaria control** *Medical and Veterinary Entomology* 2000, **14:**345-354.

2. Conteh L, Sharp BL, Streat E, Barreto A, Sundragasen K: **The cost and cost-effectiveness of malaria vector control by residual insecticide house-spraying in southern Mozambique: a rural and urban analysis.** *Tropical Medicine and International Health* 2004, **9:**125-132.

3. Goodman C, Coleman P, Mills A: **Economic Analysis of Malaria Control in Sub-Saharan Africa.** pp. 185. Geneva: Global Forum for Health Research; 2000:185.

4. Walker K: **Cost-comparison of DDT and alternative insecticides.** *Medical and Veterinary Entomology* 2000, **14:**345-354.
