# Supplementary material for: Improving the cost-effectiveness of IRS with climate informed health surveillance systems
Source: Malar J. 2008 Dec 24;7:263. doi: 10.1186/1475-2875-7-263 (PMC2639594; doi:10.1186/1475-2875-7-263)
Supplement: Additional File 2 — Cost of Hwange District, Zimbabwe IRS programme. Detailed results of Hwange district IRS programme costing analysis including unit cost data. [file 1475-2875-7-263-S2.docx]

# Appendix 2: Cost of Hwange District, Zimbabwe IRS programme

## Description of the IRS programme in Hwange District

IRS programme activity begins with chemical procurement administered at the central level in Harare. Once the chemicals have been delivered to Harare they are stored at the Blair Research Centre until the Provinces send trucks to collect them. Chemicals are then stored at the Provincial level to await collection or distribution to the Districts.

The recruitment and training of the spray teams is organised at district level and carried out by experienced spray men or the District Environmental Health Officer (DEHO). Spray men are employed on a casual basis for three months and training takes place at the beginning of this time for a period of up to two weeks. Spray teams and all the necessary equipment are transported to the initial spray sites by a truck driven by the team leader. In Hwange district, two teams of 17 men consisting of 16 spray men and one “warner”, carry out the spray programme. A “warner” goes ahead of the team to warn villagers that the team is coming, ensure that they have emptied their homes of furniture and fetched sufficient water to dilute the chemical. Spray men follow and carry out the spraying. The teams travel on foot or if long distances are involved they will be picked up by a truck and driver and transported to the next village. The teams carry sufficient chemical for the days spraying with them in its undiluted form. Workers camp overnight in between each days work and a guard is hired to watch over the campsite and chemicals while the spray teams are at work. Programme supervisors were employed at provincial level and given access to a vehicle to oversee the quality of the spraying. Prior to the start of IRS, community leaders were sensitised by the environmental health technician (EHT) of the local rural health centres (RHC). The community leaders then sensitised households.

## Costing methodology

Data was collected from a variety of sources (commitment registers, invoices, requests for funds to central office, log books and other records) at the District, Provincial and National levels. This information was combined with qualitative information gained through formal and informal meetings with staff involved in the programme and used to construct a detailed estimate of the total cost of the programme at district level.

Data on the quantity and cost of chemicals was collected from the Department of Epidemiology and Disease Control (EDC), at the Central level Ministry of Health and Child Welfare (MoH&CW) and from the District level for cross checking.

Data on provincial level labour/staff was obtained from the provincial level and allocated to Hwange District based on the number of spray teams in Hwange as a proportion of those in the whole district (2 out of 11). Information was also obtained on district level labour/staff hired. Provincial and District level labour costs were summed to give total labour costs.

Information on the type and quantity of equipment used, and its useful life were gathered by discussions with the DEHO and the Provincial Environmental Health Officer (PEHO). This information was used to construct a list of the equipment needed to carry out the spray programme for Hwange District for each spray team. This was then multiplied by the number of spray teams (two) to give the total equipment used. Capital items were annualised using a discount rate of 5% over their useful life.

The vehicles used in the spray programme are held at a provincial vehicle pool and have to be booked out for use during spray programme activities. The distance travelled is recorded and the district pays the vehicle pool a fixed amount per kilometre, depending on the type of vehicle. This fee comes out of the district field vote therefore information on the distance travelled and amount spent on transport (excluding the cost of drivers which is included under labour) was available from the DEHO. The use of this figure in the costing assumes that it accurately reflects the economic cost of purchase, maintenance and running the vehicles (i.e. imputed rent).

The costs of community education were assessed during visits to three rural health centres (RHC) in the Hwange district. The environmental health technician (EHT) in each RHC was interviewed to assess the length of time spent on community education prior to the spray programme and any other costs associated with the activity. The cost of EHT time was calculated using data on the wage for EHTs, which was obtained from the provincial level. The average cost from these RHC was then calculated and multiplied by the number of RHC in the district.

Administration and organisation of the spray programme will incur costs at the district, provincial and national (MoH&CW) levels. For example these will include the cost of staff time at the MoH&CW incurred during the tender process for the chemicals, the organisation of their distribution and any meetings and administration carried out by staff at district, provincial or national level. Given that these activities are carried out for the whole of the country the proportion allocated to Hwange district is likely to be negligible. They were therefore excluded. Storage costs are also incurred while the chemicals are at the central level waiting to be collected by the provinces and at the provincial level whilst awaiting collection by the districts. A proportion of the running and capital costs of the building in which these activities take place could therefore also have been included in the analysis however, these costs were considered to be minimal, particularly in relation to the substantial amount of effort that would be needed to cost them and they were therefore excluded from the analysis. Furthermore, it was not necessary to include overhead and capital costs of buildings in the analysis since all other activities would be carried out from camps (e.g. spraying activities) which have been costed or in the community (e.g. community education) which is excluded from the provider perspective.

All cost data was collected in the actual currency used for each transactions (all in Zimbabwe Dollars apart from chemical purchase) and converted into US$ for the presentation of results. All results are given in 1999 US$.

Data on the number of structures sprayed and estimated population protected was obtained and used to calculate a cost per person protected.

## Costing results

The IRS programme used both Deltamethrin (K-Othrin’®) and lambda-cyhalothrin (Icon’®). The cost and quantity of each chemical procured was used to calculate the cost per charge for each chemical in US$ and the results are shown in Table 8. The total expenditure on chemicals for the 1998/99 malaria season in Hwange district was US$38,023.

The total labour cost (excluding community education activities) for the Hwange District spray programme was US$1947. Spraymen accounted for the largest amount of labour costs US$1418.95 (74%). The results of the labour costing are shown in Table 9.

The total cost of equipment per spray team with capital items was US$2559. Two teams were used to cover Hwange District, therefore the total cost of equipment was US$5118. The unit cost of each item was obtained from a purchase order at the provincial medical office with the exception of the pump spares/maintenance kits for which no information could be found so US$10 was used (Hudson Price Catalogue, cited in Goodman et al. 1999). The detailed cost and quantity of equipment is shown in Table 10.

The vehicle used for supervisory purposes covered a distance of approximately 2500km at a cost of US$0.148/km and the vehicle used to transport the spray teams travelled a distance of approximately 7500km at a cost of US$0.145. The total cost of these two activities’ transport was US$1459.

It was estimated that the EHT spent an average of two days organising, preparing for and attending the meeting of community leaders prior to the spray programme. The meeting would normally take place outside in a communal meeting area around or near to the health centre. No transport or subsistence costs would therefore be imposed on the provider and the only cost was the EHTs time. For the 16 government run RHC in Hwange district the total cost of community education in Hwange district was therefore US$518.

Table 8 Chemical costs (US$1999)

| Type of Chemical | Chemical Quantity | | Cost Per Litre/Kg | Chemical Unit Cost/Charge | Total Chemical Cost |
| --- | --- | --- | --- | --- | --- |
|  | In Litres/Kg | In Charges* |  |  |  |
| Icon | 386.69 | 6187 | 85.00 | 5.31 | 32852.97 |
| K-Othrin | 60.00 | 1000 | N/A | 5.17 | 5170.00 |
| Total | N/A | 7187 | N/A | N/A | 38022.97 |

* A charge of each chemical is the amount that must added to a fixed amount of water (10 litres) to produce a solution of the recommended concentration. A charge of K-Othrin is 60ml and a charge of Icon is 62.5 grams.

Table 9 Labour Costs for 1998/99 Hwange District Spray Programme

| Occupation | Quantity | Monthly or Daily Wage (US$) | Number of Month (M) or Days (D) Hired | Total Cost (US$) | % of Total Labour Costs |
| --- | --- | --- | --- | --- | --- |
| Spraymen | 30 | 15.77 (M) | 3 (M) | 1418.95 | 74 |
| Warners | 2 | 16.66 (M) | 3 (M) | 99.97 | 5 |
| Drivers | 1 | 58.14 (M) | 3 (M) | 174.41 | 9 |
| Guards | 2 | 0.94(D) | 66(D) | 124.04 | 6 |
| Supervisors | 2 | 1.83(D) | 12(D) | 43.85 | 2 |
| Team Leaders | (2*2/11)=0.36 | 78.31(M) | 3(M) | 85.43 | 4 |
| TOTAL | N/A | N/A | N/A | 1946.65 | 100 |

Table 10 Quantity, Useful life and Total Cost of Equipment used by a Spray Team

| Item Use Category | Item | Number per Team | Estimated Useful Life (Years) | Total economic cost per year US$^[[1]](#footnote-2)^ |
| --- | --- | --- | --- | --- |
| Spraying | Spray Pumps | 15 | 5 | 388.87 |
|  | Pump Spares/Maintenance kit | 2 | 2 | 10.67 |
|  | Pump Hose (meters) | 16 | 1 | 16.71 |
|  | Haver Sack/Knapsacks | 15 | 2 | 16.85 |
|  | Shifting Spanner | 1 | 2 | 0.70 |
| Mixing Equipment | Galvanised Buckets | 15 | 5 | 17.54 |
|  | Strainers | 15 | 5 | 1.09 |
| Protective Gear | Overalls | 35 | 1 | 240.64 |
|  | Rubber Gloves | 15 | 2 | 5.26 |
|  | Farm Shoes/ Rubber Boots (Pairs) | 15 | 2 | 758.07 |
|  | Floppy Hats | 15 | 2 | 10.68 |
|  | Rain Coats | 15 | 5 | 15.55 |
|  | Face Shields | 15 | 5 | 4.52 |
|  | Respirator Cartridges | 15 | 1 | 38.76 |
|  | Soap Bars | 120 | 1 | 18.79 |
| Camping Equipment | 4 Men Tents | 5 | 5 | 696.36 |
|  | Roof Tents | 1 | 5 | 108.52 |
|  | Ground Sheeting (Sq M) | 20 | 2 | 21.06 |
|  | Hurricane Lamps | 5 | 2 | 4.77 |
|  | Torches | 5 | 2 | 3.51 |
|  | Water Cooling Bags | 1 | 1 | 0.78 |
|  | Hoe | 1 | 5 | 0.12 |
|  | Axe | 1 | 5 | 1.11 |
|  | Pick | 1 | 5 | 1.02 |
|  | Pick Handle | 2 | 1 | 1.67 |
|  | Shovels | 2 | 5 | 1.81 |
|  | Stretcher Bed Frames | 18 | 5 | 20.62 |
|  | Stretcher Bed Canvas | 18 | 2 | 96.02 |
|  | Folding Chairs | 18 | 5 | 13.13 |
|  | Chair Canvasses | 18 | 2 | 30.32 |
|  | Folding Tables | 5 | 5 | 4.22 |
| Miscellaneous | Hessian Sacking (Sq M) | 10 | 1 | 5.22 |
|  | Exercise Books | 20 | 1 | 1.57 |
|  | Ball Pens | 20 | 1 | 2.61 |
| TOTAL COST PER TEAM | | N/A | N/A | 2559.17 |
| TOTAL COST HWANGE DISTRICT | |  |  | 5118.34 |

1. Capital items discounted at 5% [↑](#footnote-ref-2)
